# Rare case of exostosin 1/exostosin 2-related membranous lupus nephritis concomitant with dual ANCA- and anti-GBM antibody-associated crescentic glomerulonephritis effectively diagnosed by mass spectrometry: a case report

**DOI:** 10.1186/s12882-023-03268-1

**Published:** 2023-07-24

**Authors:** Takuya Yamazaki, Haruka Takahashi, Kazuhiro Takeuchi, Emi Sakamoto, Kenta Tominaga, Syun Sakurabayashi, Tetsuya Abe, Takashi Sano, Yukihiro Wada, Naomi Kuwahara, Akira Shimizu, Yasuo Takeuchi

**Affiliations:** 1 Department of Nephrology, Kitasato University School of Medicine, 1-15-1 Kitasato, Minami-Ku, SagamiharaKanagawa, 252-0374 Japan; 2 Omigawahimawari Clinic, Chiba, Japan; 3 Department of Analytic Human Pathology, Nippon Medical School, Tokyo, Japan; 4 Aihara Hospital, Kanagawa, Japan

**Keywords:** Lupus nephritis, ANCA-associated vasculitis, Anti-GBM glomerulonephritis, Mass spectrometry, Exostosin ½

## Abstract

**Background:**

Recent developments in mass spectrometry (MS) have revealed target antigens for membranous nephropathy (MN), including phospholipase A2 receptor and exostosin 1/exostosin 2 (EXT1/2). EXT1/2 are known antigens of autoimmune disease-related MN, especially membranous lupus nephritis. We describe the case of an elderly man who developed nephrotic syndrome followed by progressive renal dysfunction.

**Case presentation:**

A 78-year-old man presented with rapidly progressive renal dysfunction with proteinuria and hematuria. Three years previously, he had developed leg edema but did not receive any treatment. Laboratory tests showed elevated anti-nuclear antibody (Ab), anti-dsDNA Ab titer, and hypocomplementemia, indicating systemic lupus erythematous. Myeloperoxidase anti-neutrophil cytoplasmic Ab (ANCA) and anti-glomerular basement membrane (GBM) Ab were also detected. The renal pathologic findings were compatible with crescentic glomerulonephritis (GN), whereas non-crescentic glomeruli exhibited MN without remarkable endocapillary or mesangial proliferative change. Immunofluorescence microscopy revealed glomerular IgG, C3, and C1q deposition. All IgG subclasses were positive in glomeruli. Anti-PLA2R Ab in serum was negative. MS analysis was performed to detect the antigens of MN, and EXT1/2 was detected in glomeruli. Therefore, we reached a diagnosis of membranous lupus nephritis concurrent with both ANCA-associated vasculitis and anti-GBM-GN. The simultaneous occurrence of these three diseases is extremely rare.

**Conclusions:**

This is the first report of EXT1/2-related membranous lupus nephritis concurrent with ANCA-associated vasculitis and anti-GBM-GN. This case demonstrates the usefulness of MS in diagnosing complicated cases of MN.

**Supplementary Information:**

The online version contains supplementary material available at 10.1186/s12882-023-03268-1.

## Background

Systemic lupus erythematous (SLE) is a well-known disease that occurs most commonly in young women. In recent years, however, there have been increased reports of elderly-onset SLE, which has a lower female-to-male ratio and does not exhibit typical symptoms such as butterfly erythema [1, 2].

Although the concurrence of SLE and antineutrophil cytoplasmic antibody-associated vasculitis (ANCA-AV) is rare, it is reported as an overlapping syndrome [3–5]. Patients with ANCA-positive SLE are known to show high pathological activity in renal biopsy and a poor renal prognosis [6, 7].

Anti-glomerular basement membrane glomerulonephritis (GBM-GN) is well known as the cause of rapidly progressive GN. The antigen of anti-GBM antibody (Ab) is α3(IV)NC1 domain of type-IV collagen. The characteristic pathological feature is crescentic GN with large diffuse cellular crescents in a similar time phase [8]. Patients with ANCA-AV are likely to develop anti-GBM-GN [9, 10]. To the best of our knowledge, the concomitance of the three diseases of SLE, ANCA-AV, and anti-GBM-GN is extremely rare.

Membranous nephropathy (MN) is conventionally divided into primary and secondary forms depending on the presence or absence of background diseases such as cancer, autoimmune disease, and drug use. However, antigens of MN are now being successively discovered, which is partly due to remarkable advances in mass spectrometry (MS) and laser microdissection [11]. Therefore, it is now recommended that MN should be classified according to the pathogenic antigen. Exostosin 1 and 2 (EXT1/2) are recently reported antigens of autoimmune disease-related secondary MN [11, 12].

In this report, we describe the case of an elderly male SLE patient with myeloperoxidase (MPO) ANCA and anti-GBM Ab, in whom crescentic GN and MN were apparent in renal biopsy tissue. MS analysis performed for antigen identification detected EXT1/2 in glomeruli. To the best of our knowledge, this is the first reported case of EXT1/2 -related membranous lupus nephritis accompanied by ANCA-AV and anti-GBM-GN.

### Case presentation

The patient was a 78-year-old Japanese man who had noticed edema in both legs three years earlier. The edema had worsened over the 12 months preceding his admission to our hospital, and he had developed malaise and anorexia. He was referred to our hospital one week after a blood test revealed high urea nitrogen (32.9 mg/dL) and serum creatinine (sCr) (2.91 mg/dL). On admission, blood pressure was 151/89 mmHg and heart rate was 83 beats/min with normal heart function. Pitting edema was observed in both legs. No other abnormalities were observed, including of the skin. Urinalysis showed proteinuria of 6.11 g/gCr and hematuria with glomerular hematuria and various types of urinary cast (Table 1). The sCr level worsened to 4.25 mg/dL, indicating rapidly progressive renal dysfunction. Serum C reactive protein was 7.57 mg/dL. Immunological laboratory tests showed a low level of serum C3 and high levels of anti-nuclear Ab (80 titer), anti-dsDNA Ab (220 IU/mL), MPO ANCA (9.7 U/mL), and anti-GBM Ab (208.0 U/mL) (Table 1). These data suggested a diagnosis of rapidly progressive renal dysfunction due to the concurrence of SLE, ANCA-AV, and anti-GBM-GN. Chest and abdominal computed tomography (CT) (supplemental Fig. 1a and b) revealed only ascites, which was possibly related to the rapidly progressing renal dysfunction.

**Table 1:** Laboratory data on admission

< Urinalysis >		< Blood cell count >			< Immunology >		
Gravity	1.014	WBC	5700	/μL	CRP	7.57	mg/dL
pH	6.5	Hb	9.4	g/dl	IgG	2186	mg/dL
Blood	(3 +)	Plt	24.9	× 10^4^ /μL	IgA	375	mg/dL
protein	(3 +)	< Blood chemistry >			IgM	54	mg/dL
	6.11 g/g・Cr	TP	6.4	g/dL	C3	63	mg/dL
24-h proteinuria	2.0 g/day	Alb	2.1	g/dL	C4	23	mg/dL
< Urinary sediment >		T-Bil	0.4	mg/dL	CH50	27	U/mL
RBC	> 100/HPF	LDH	261	U/L	ANA	× 80	Titer
Fatty cast	( +)	BUN	46.5	mg/dL	Anti dsDNA	220	IU/mL
Waxy cast	( +)	Cr	4.25	mg/dL	MPO ANCA	9.7	U/mL
dysmorphic RBC	( +)	eGFR	11	ml/min/1.73m^2^	PR3 ANCA	< 1.0	U/mL
		Na	135	mEq/L	Anti GBM Ab	208	U/mL
		K	4.5	mEq/L	Anti PLA2R	< 0.7	negative
		Cl	96	mEq/L			
		Ca	8.2	mg/dL			
		P	4.7	mg/dL

**Fig. 1:**
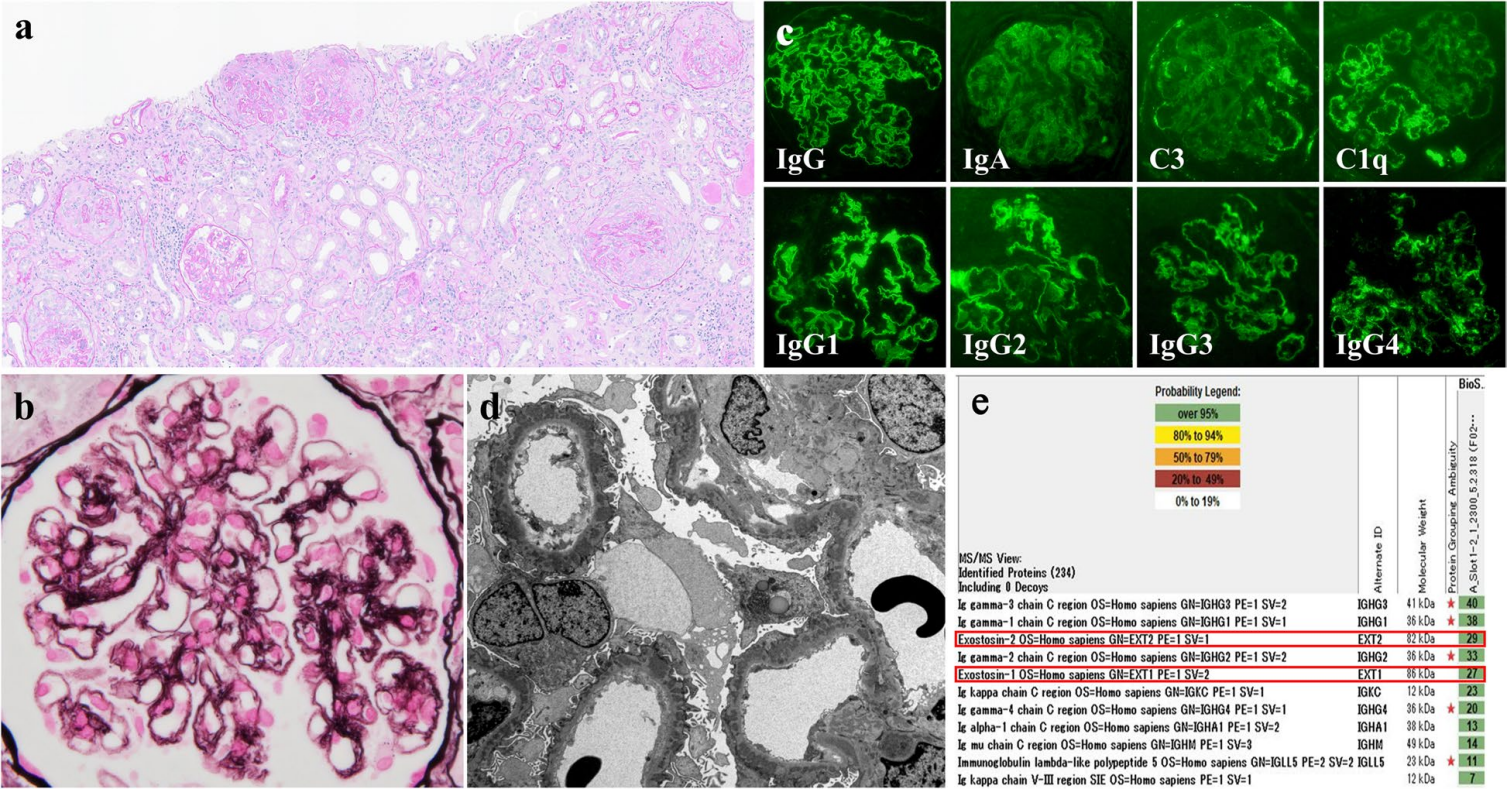
Pathological findings in the present patient. **a** and **b:** Light microscopic images of renal biopsy tissue. **a** PAS staining (× 10). **b** PAM staining (× 40). **c** Immunofluorescence analysis of renal biopsy tissue. IgG was linear-positive for glomerular basement membrane and partially granularly positive for outer side of glomerular basement membrane. C3 and C1q were granularly positive for glomerular basement membrane. **d** Electron microscopic findings of renal biopsy tissue. A markedly large number of subepithelial electron-dense deposits (EDD) were observed, as well as small amount of irregular mesangial EDD. Basement membrane was thickened. **e** Mass spectrometry analysis using glomeruli from formalin fixed paraffin embedded tissue section by laser microdissection. Exostosin 1 and 2 were detected (red outlines). PAS, Periodic Acid Schiff; PAM, Periodic Acid-Methenamine silver

A renal biopsy was performed to investigate the cause of renal dysfunction. Light microscopic findings showed 23 cellular crescents, 8 fibrocellular crescents, 2 fibrous crescents, and 8 with global sclerosis in a total of 58 glomeruli (Fig. 1a). The GBM in glomeruli with preserved structure was thickened, and spikes and stippling were observed (Fig. 1b). Interstitial cell infiltration with edema and fibrosis were also observed. Immunofluorescence (Fig. 1c) showed IgG deposits at the capillaries, mainly in a linear pattern, and a partially granular pattern at the outer side of the GBM. C3, and C1q also showed granular patterning at the capillaries. IgG subclass staining revealed that IgG1, IgG2, and IgG3 were linearly positive, whereas IgG4 was granularly positive at the capillaries (Fig. 1c). Electron microscopy (Fig. 1d) revealed obvious subepithelial electron-dense deposits (EDD) and a small amount of irregular mesangial EDD was also observed. The above results suggested a diagnosis of crescentic GN and membranous nephropathy due to lupus nephritis, ANCA-AV, and anti-GBM-GN.

M class phospholipase A2 receptor (PLA2R) antibodies in serum was negative. Immunohistochemical staining for PLA2R was also negative (data not shown). In addition, MPO staining was negative in the glomeruli (supplemental Fig. 2a). To identify the antigens of MN and further clarify the pathogenesis of this disease, the glomeruli were extracted by laser microdissection and MS analysis was performed. Both EXT1 and EXT2 were detected (Fig. 1e), but PLA2R and MPO were not detected in MS. Further confirming MS findings, IHC for EXT1 and EXT2 staining were positive in the glomerular capillary wall (supplemental Fig. 2b and c).

A steroid pulse of methylprednisolone 500 mg/day was administered for three days followed by 40 mg of oral prednisone. A total of seven plasmaphereses were performed from day 4 after admission; however, the patient’s renal function did not recover and maintenance hemodialysis was required from day 33 after admission.

## Discussion and conclusions

It is well known that SLE is the most common in women aged 20–40 years. In young-onset SLE patients, the male:female ratio is 1:10. Regarding age at the onset of SLE, approximately 20% of patients are 50–69 years of age, and 8% of patients are aged ≥ 70 years[2]. Another study has reported that 20% of cases are diagnosed at an advanced age, for which the male:female ratio is 1:2.5 [13]. In the present case report, the elderly male patient had low complement level, high anti-nuclear Ab, dsDNA Ab, and nephritis, and was diagnosed with SLE according to the 2019 EULAR/ACR classification criteria [14]. Most noticeably, EXT1/2 were detected by MS. This is the first report to describe detection of EXT1/2 in an SLE patient with ANCA-AV and anti-GBM-GN. It has been reported that over 80% of EXT1/2-positive patients had an autoimmune disease such as SLE or Sjögren syndrome [15]. Therefore, the membranous nephritis lesion in the present case was most likely class V lupus nephritis. In this case, only IgG4 was deposited in granular pattern. Each of all four IgG subclass were reported to be detected in EXT1/EXT2-associated MN [15]. IgG4 appeared to be the causative antibody for MN in our case. EXT1 and EXT2 are glycosyltransferases that exist as a heterodimeric copolymerase complex responsible for synthesis of the heparan sulfate chain in the GBM [16]. The pathological class of lupus nephritis in EXT1/2-positive patients had been reported mainly as “pure class V” [15]. In a larger number of cohort studies, however, the same research group subsequently found no difference regarding EXT1/2-positivity between pure class V lupus nephritis and class III/IV + V lupus nephritis [17]. Retrospective cohort studies of patients with membranous lupus nephritis revealed that EXT1/2-positive patients had higher levels of proteinuria, and have a better renal prognosis compared with patients with EXT1/2 negative lupus nephritis [17, 18]. In our case, there is a possibility that the patient had class IV and V lupus nephritis. However, due to the absence of subendothelial deposits on electron microscopy and wire loop lesions on light microscopy, we believe that our patient had class V lupus nephritis alone. As his edema had been apparent for three years, MN due to class V lupus nephritis might have developed long ago.

In this patient, MS analysis was effective in detecting EXT1/2. Another option is to use immunostaining to find antigens for membranous nephropathy. Immunostaining is a very effective method when antigens are presumed. However, because the number of reported antigens is continuing to increase, and therefore the number of antibodies for staining is also increasing. MS enables comprehensive observations that are obtained simultaneously. In addition, MS is the only method for identification of new antigens; and as in the present case, may help to elucidate the pathophysiology. Previous studies have reported association of ANCA-AV with MN and of anti-GBM-GN with MN [19, 20]. In ANCA-AV related MN, it has been reported that MPO is an antigen of MN [19]; in another study, 66.7% of anti-GBM-GN-related MN cases were reported to be positive for PLA2R [20]. MN due to these diseases was a possibility in our patient because he was positive for both ANCA and anti-GBM Ab; however, as he was EXT1/2-positive, we strongly suspected MN due to lupus nephritis. In addition, because PLA2R staining and MPO staining were both negative in our case, a diagnosis of PLA2R-related MN or ANCA-AV-related MN was unlikely.

Almost half of the glomeruli observed in renal biopsy tissue were composed of cellular crescents in a similar time phase. A small number of different time phase crescents, fibrocellular and fibrous crescents, were also observed. Crescents observed in ANCA-AV are usually more variable in time phase compared with those in anti-GBM-GN, in which all crescents tend to show the same stage of activity and chronicity [8, 21]. The present findings indicated that ANCA-AV could have developed secondary to SLE, followed by anti-GBM-GN, resulting in rapidly progressive renal dysfunction. In other words, fibrocellular and fibrous crescents and global sclerosis could be associated with ANCA-AV, and cellular crescents of a similar time phase could be caused by anti-GBM-GN. In this case, IgG1, IgG2 and IgG3 were deposited in linear pattern. All four IgG subclass are known to be detected as an anti-GBM-Ab. Further, IgG1 and IgG3 are reported to have a pivotal role in the initiation and progression of anti GBM-GN [22]. The IgG1 is reported as the most important contributor for the severity of renal damage [22]. MPO ANCA was positive in this case, implying an overlap syndrome of SLE and ANCA-AV. Li et al. reported an ANCA positivity rate in lupus nephritis of 3.69% [6]. Patients with both SLE and MPO ANCA have a higher incidence of necrotic lesions and crescent formation in renal biopsy, although the rate of crescent formation was not significantly different [7]. In contrast, a relationship between anti-GBM-GN and ANCA-AV has been reported, in which 30%–38% of anti-GBM Ab-positive patients were positive for ANCA. In contrast, 7.5%–14% of ANCA-positive patients were positive for anti-GBM Ab [9, 10]. Although the details of the mechanism remain unclear, it is thought that GBM injury due to ANCA-AV uncovers the antigen, which subsequently acts as a trigger for development of anti-GBM-GN [23]. The findings of these reports make us more inclined to consider that anti-GBM-GN developed secondary to ANCA-AV in the present patient. Levy et al. reported on treatment response in patients with both anti-GBM-GN and ANCA-AV [24]. Our patient needed permanent hemodialysis, which is in agreement with previous reports that patients with both ANCA-AV and anti-GBM-GN were more likely to require hemodialysis compared with those who were positive for anti-GBM Ab alone, and had an overall poor prognosis [24, 25].

In conclusion, we describe a very rare case of EXT1/2-related membranous lupus nephritis concomitant with dual ANCA- and anti-GBM Ab-associated crescentic glomerulonephritis. MS enabled to characterize the causal antigens of MN, which helped us designing the therapeutical plan and follow-up of the patient. Despite the complicated renal findings in the present patient, MS led to the diagnosis of a single pathophysiology of membranous lupus nephritis with subsequent ANCA-AV and anti-GBM-GN. Accordingly, we recommend analysis by MS for complicated cases of MN.

## Supplementary Information

**Additional file 1: Supplemental Figure 1.** **a** Computed tomography (CT) of the chest. No abnormalities such as alveolar hemorrhage or interstitial pneumonia were noted. **b** Abdominal CT scan. No abnormalities other than ascites were observed.

**Additional file 2: Supplemental Figure 2.** **a** Myeloperoxidase (MPO) staining using green color (FITC). There was no positive signal in glomeruli. **b** and **c**: Exostosin (EXT)1 and EXT2 staining using red color (Alexa 568), respectively, are both positive in glomerular capillary wall.

## Data Availability

All data generated or analyzed during this study are included in this published article.

## Abbreviations

Ab: Antibody
ANCA: Antineutrophil cytoplasmic antibody
ANCA-AV: Antineutrophil cytoplasmic antibody-associated vasculitis
dsDNA: Double stranded deoxyribonucleic acid
EDD: Electron dense deposit
EXT1: Eostosin 1
EXT2: Exostosin 2
GBM: Glomerular basement membrane
GBM-GN: Anti-glomerular basement membrane glomerulonephritis
MN: Membranous nephropathy
MPO: Myeloperoxidase
MS: Mass spectrometry
PLA2R: Phospholipase A2 receptor
sCr: Serum creatinine
SLE: Systemic lupus erythematous
