# Supplementary figures and images for: Rare case of exostosin 1/exostosin 2-related membranous lupus nephritis concomitant with dual ANCA- and anti-GBM antibody-associated crescentic glomerulonephritis effectively diagnosed by mass spectrometry: a case report
Source: BMC Nephrol. 2023 Jul 24;24:218. doi: 10.1186/s12882-023-03268-1 (PMC10364369; doi:10.1186/s12882-023-03268-1)

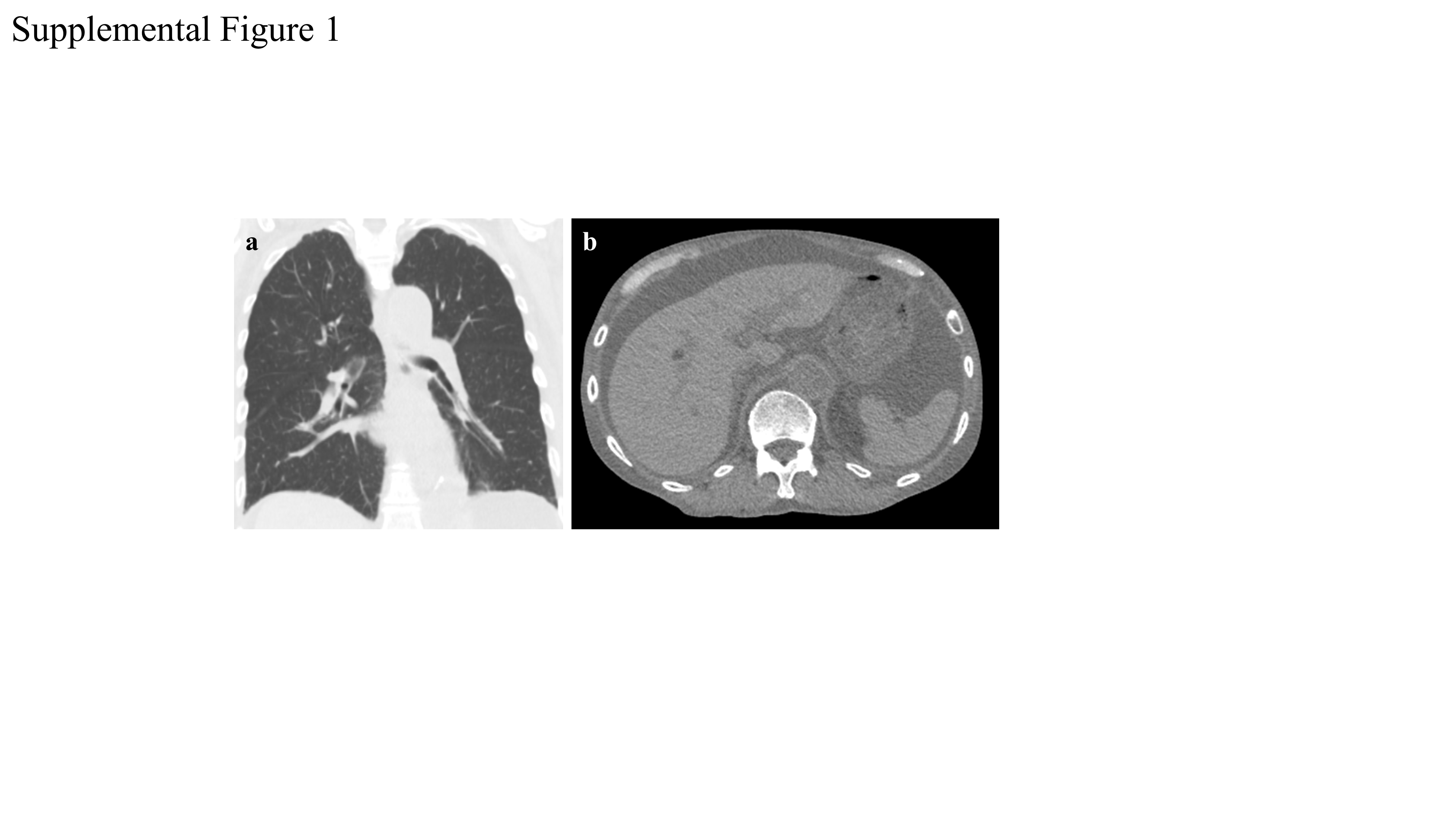

Supplement: Supplementary file 1 — Additional file 1: Supplemental Figure 1. a Computed tomography (CT) of the chest. No abnormalities such as alveolar hemorrhage or interstitial pneumonia were noted. b Abdominal CT scan. No abnormalities other than ascites were observed. [file 12882_2023_3268_MOESM1_ESM.tif]

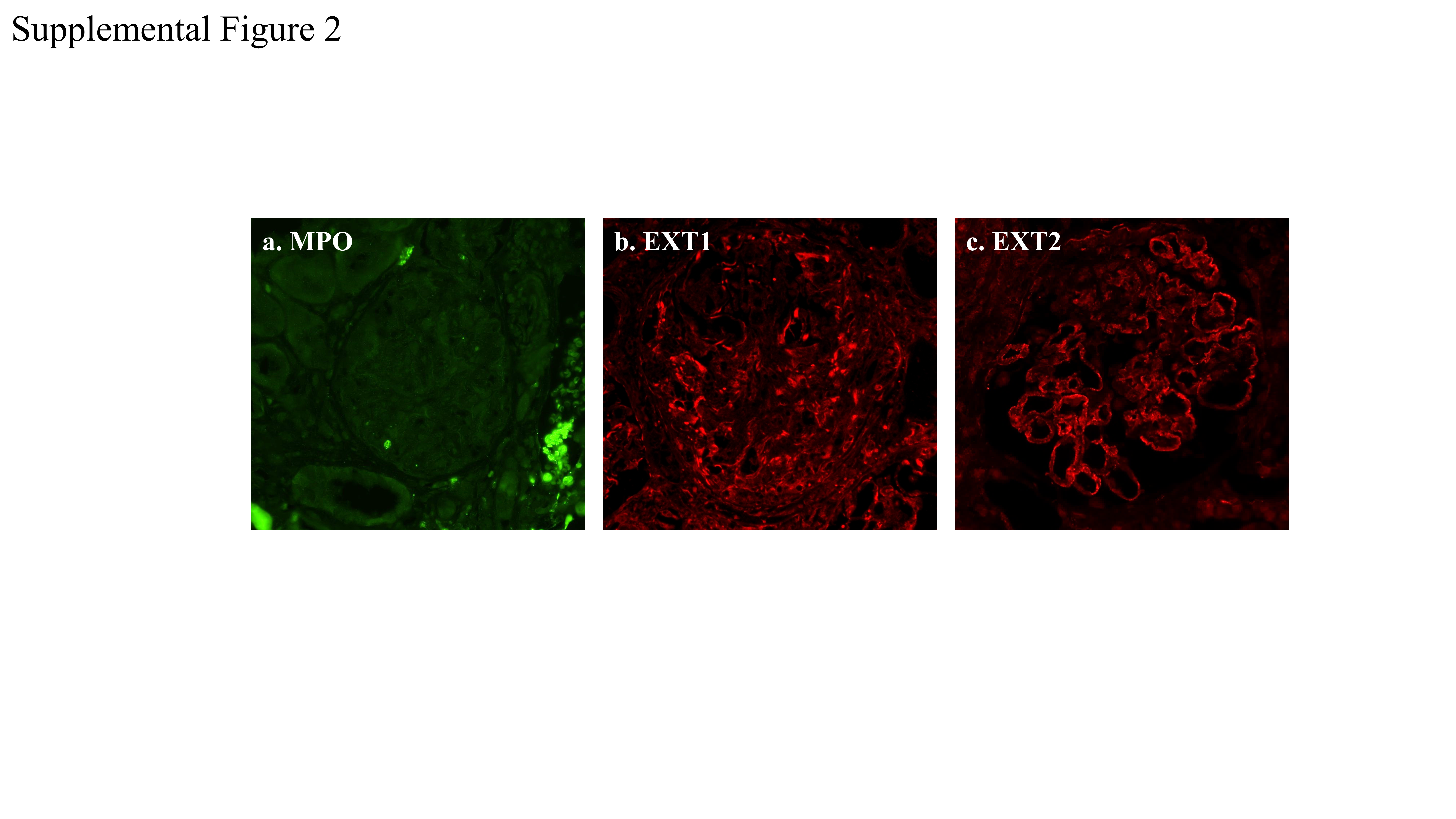

Supplement: Supplementary file 2 — Additional file 2: Supplemental Figure 2. a Myeloperoxidase (MPO) staining using green color (FITC). There was no positive signal in glomeruli. b and c: Exostosin (EXT)1 and EXT2 staining using red color (Alexa 568), respectively, are both positive in glomerular capillary wall. [file 12882_2023_3268_MOESM2_ESM.tif]
